# Impact of fast-track discharge from cardiothoracic intensive care on family satisfaction

**DOI:** 10.1186/2197-425X-3-S1-A356

**Published:** 2015-10-01

**Authors:** PC Sivadasan, AS Omar, M Gul, R Taha, AK Tuli, R Singh

**Affiliations:** Hamad Medical Corporation, Cardiothoracic Surgery-Heart Hospital, Doha, Qatar; Faculty of Medicine, Critical Care Medicine, Beni Suef, Egypt; Hamad Medical Corporation, Medical Research Center, Biomedical Statistics, Doha, Qatar

## Introduction

Dissatisfaction with the intensive care unit may threaten medical care. Clarifying treatment preferences can be useful in these settings, where physician direction may influence decision making and therefore medical treatment. This study aimed to evaluate whether fast-track discharge from intensive care units affects the satisfaction of family members [1].

## Objectives

Evaluate the satisfaction of the family members and to assess its determinants in Qatari intensive care related to fast- track discharge [2] from the ICU.

## Methods

We used a single-center non-randomized trial, with all eligible family

members involved. To evaluate family satisfaction, we used the Society of Critical Care Family Needs Assessment questionnaire (SCCMFNAQ). We hypothesized that those discharged within 24 hours of intensive care unit admission and their families would have higher levels of satisfaction. Patients were scored using the therapeutic interventions scoring system (TISS) and additive EuroSCORE.

## Results

Two-hundred fifty-five family members were enrolled. The mean patient age was 53 years, and 92% were male. The median satisfaction level among family member was 17.9 (range 14-31). Patients were divided into two groups, one receiving fast-track discharge (115 patients), and one whose members stayed longer (139 patients). The overall satisfaction was affected significantly by quality of the delivered care and dissatisfaction increased by lack of comfort in hospital settings, including the waiting room. No significant differences were seen between the two groups for overall satisfaction (p = 0.546) and individual components of the questionnaire. Higher satisfaction was linked to higher levels of education among family members (p = 0.045) and information being relayed by a senior physician p = 0.03 (two-tailed test).

## Conclusions

Fast-track discharge from intensive care did not influence family satisfaction as hypothesized. Satisfaction relied on family members´ level of education and the level of seniority of the physician relaying information.

## Grant Acknowledgment

To all members of cardiothoracic surgery department and medical research center, Hamad medical corporation, Qatar

**Figure 1:**
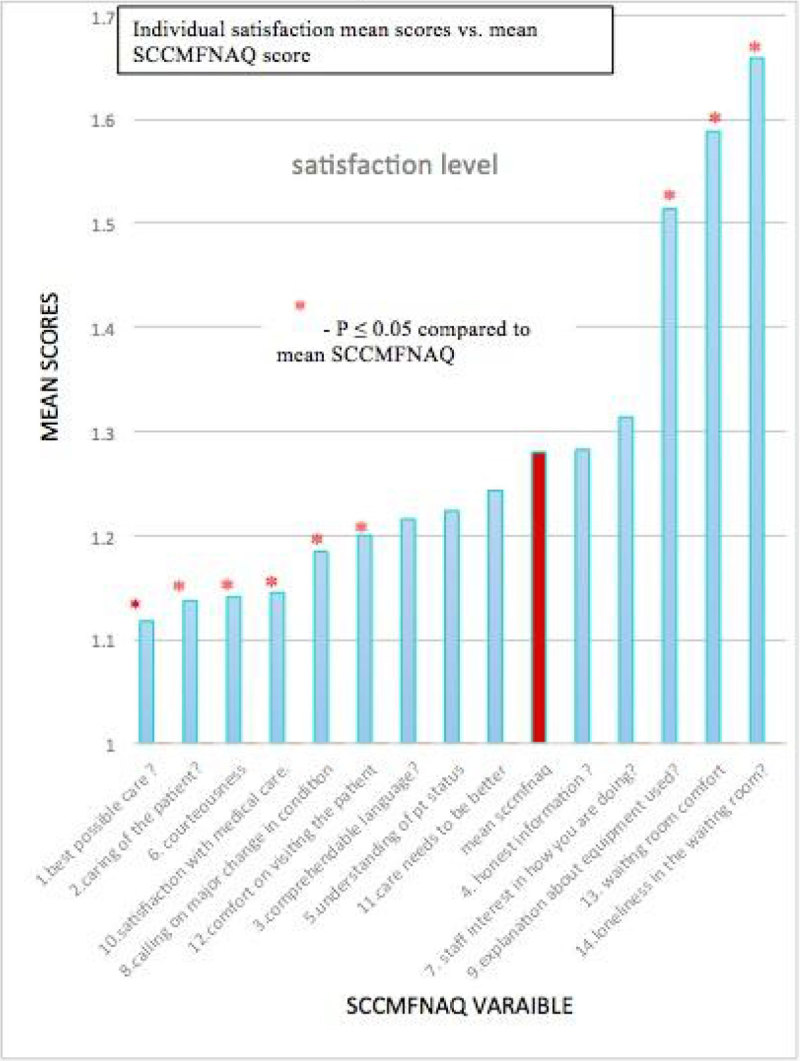
Figure 1
